# Sex and age do not modify the association between glucocorticoids and bone mineral density in patients with rheumatoid arthritis: a cross-sectional study

**DOI:** 10.1186/s13075-023-03083-x

**Published:** 2023-06-07

**Authors:** Andriko Palmowski, Zhivana Boyadzhieva, Sabrina M. Nielsen, Burkhard Muche, Sandra Hermann, Maarten Boers, Henning Bliddal, Robin Christensen, Edgar Wiebe, Frank Buttgereit

**Affiliations:** 1 Department of Rheumatology and Clinical Immunology, Charité – Universitätsmedizin Berlin, Berlin, Germany; 2 Section for Biostatistics and Evidence-Based Research, The Parker Institute, Bispebjerg and Frederiksberg Hospital, Copenhagen, Denmark; 3 Department of Clinical Research, Research Unit of Rheumatology, University of Southern Denmark, Odense University Hospital, Odense, Denmark; 4 Department of Epidemiology & Data Science, Amsterdam University Medical Centers, Vrije Universiteit, Amsterdam, Netherlands

**Keywords:** Rheumatoid arthritis, Osteoporosis, Glucocorticoids, Prednisone, Effect modifier, Age, Sex, Interaction

## Abstract

**Background:**

It is unclear whether sex or age modify the association of glucocorticoid (GC) use with reduced bone mineral density (BMD) in patients with rheumatoid arthritis (RA).

**Methods:**

We studied cross-sectional data of RA patients with current or previous GC treatment in a single center cohort study (*Rh-GIOP* cohort). Our primary outcome was the minimum T-score (measured by DXA) of either lumbar spine, total femur, or femoral neck. Current GC dose was the main exposure; cumulative GC dose and cumulative duration of GC use were also assessed. Following a predefined statistical analysis plan, linear regression analyses with adjustment for confounders assessed whether the association of GC use with BMD was modified by sex (men versus women) or age (≥ 65 versus < 65 years).

**Results:**

Four hundred eighty-three patients with RA (mean age 64 ± 12 years, 80% women) were included. 33% were not currently taking GCs, 32% were treated with a dose of 5 mg/d prednisone equivalent and 11% with more than 7.5 mg/d. 23% of patients had osteoporosis by DXA (minimum T-score ≤ -2.5). The slope, i.e., the association between changes in minimum T-scores with 1 mg/d change in current GC dose, was similar in men and women (-0.07 and -0.04, respectively; difference -0.03 [-0.11 to 0.04]; *p* for interaction = 0.41). Slopes were also similar for elderly and non-elderly patients (-0.03 and -0.04, respectively; difference -0.01 [-0.06 to 0.05]; *p* for interaction = 0.77). Using cumulative dose and duration of use as exposures did not lead to substantial changes of these results.

**Conclusions:**

In our sample, the association of GC use with reduced BMD in RA was not modified by sex or age.

**Supplementary Information:**

The online version contains supplementary material available at 10.1186/s13075-023-03083-x.

## Background

Rheumatoid arthritis (RA) is a chronic inflammatory autoimmune disease with potentially negative effects on the quality of life and life expectancy of affected patients [1]. Various disease-related factors contribute to this, e.g., pain, reduced physical performance, or associated comorbidities such as osteoporosis (OP) [2].

Glucocorticoids (GCs) are widely used in RA. Current EULAR guidelines recommend GCs as a short-term treatment (“bridging therapy”), but recent research from around the globe (e.g., Europe [3, 4], China [5], Australia [6], and North America [7]) found that a substantial proportion of patients use GC for longer periods of time. GCs may cause OP, and GC-induced OP is considered the most common type of secondary OP [8, 9]. In a prior analysis of patients with RA, we found GC therapy to be only associated with bone loss when patients had both moderate or high disease activity and received ‘high’ [10] GC dosages (≥ 7.5 mg/d prednisone equivalent) [11].

In the study at hand, we sought to further analyze the associations between GCs and bone mineral density (BMD) in patients with RA by looking at potential effect modifiers: Age and sex. In other words, we hypothesized that GCs might have different effects on BMD in a) men versus women and b) in younger versus older patients. Our data analysis was planned to potentially help personalizing the management of RA regarding GC-associated OP.

## Methods

A statistical analysis plan (finalized before any analyses were conducted) was strictly adhered to and can be found in the Additional file 1: Appendix A. This article adheres to the STROBE criteria for reporting observational research [12].

### Study design and eligibility

The study at hand is a cross-sectional analysis of baseline visits of patients with RA enrolled in the Rh-GIOP cohort study, which was initiated in 2015 and is registered with clinicaltrials.gov (NCT02719314). The Rh-GIOP cohort study has received a positive vote from the local ethics committee (EA1/367/14).

Patients with inflammatory rheumatic diseases at Charité—Universitätsmedizin Berlin, a tertiary care university center, who have an indication for osteoporosis diagnostics (according to the German umbrella osteology association [DVO] guidelines) are eligible to take part. Pregnant, breastfeeding, and lactating women are excluded, as are patients unable to provide informed consent. Patients receive diagnostic procedures including a dual x-ray absorptiometry scan of lumbar spine and hip, are advised and treated according to current guidelines, and are then followed over time. Further information can be found in an earlier publication on the Rh-GIOP cohort [11].

Of note, in the analysis at hand, we only include a subpopulation of our Rh-GIOP cohort: Patients were required to have a physician diagnosis of RA, and they needed to be currently taking GCs or have a history of GC use. Patients with an identified possible other cause of a secondary OP, namely patients with a history of multiple myeloma, hyperthyroidism and hyperparathyroidism were excluded. Furthermore, we excluded patients with high GC doses that are not usually used for longer periods of time (i.e., > 15 mg/d prednisone equivalent).

### Outcomes and exposures

BMD is known to predict future fracture risk and is used by the World Health Organization to define OP. All patients in the Rh-GIOP cohort receive a BMD measurement by dual x-ray absorptiometry (GE Lunar Prodigy bone densitometer with the same operator for all patients). We defined as our main outcome the minimum observed T-score at any site. I.e., the lowest T-score was selected from either the lumbar spine, total femur, or femoral neck, whichever was lowest. This outcome was chosen as the minimum T-score of any site is recommended to guide treatment decisions in the German osteoporosis guidelines [13]. Of note, for the calculation of T-scores, the GE Lunar enCORE software with the enCORE German reference population was used with correction for ethnic origin. Femur measurements took place on both sides if possible, and left and right side measurements were averaged for analysis. In sensitivity analyses, lumbar spine and (combined) total hip T-scores were analyzed separately.

Our main exposure variable was current GC dose (in mg/d prednisone equivalent). Current GC dose is ascertained by a combination of patient self-report and chart review by the study team. Cumulative GC dose and cumulative duration of GC use were addressed in sensitivity analyses as secondary exposures of interest.

### Potential confounders

As potential confounding variables, we defined independent variables other than the exposure variable (GC) that may be correlated to the outcome (BMD) of our study. We included a variety of known and suspected confounders, namely: age (years), sex (men, women), smoking status (current, former, no smoking), body mass index (kg/m^2^), family history of osteoporotic fractures (yes/no), alcohol consumption (none, irregular/infrequent, occasional, frequent), Health Assessment Questionnaire (HAQ; score), proton pump inhibitor use (yes/no), disease duration (years), bisphosphonate use (yes/no), denosumab use (yes/no), teriparatide use (yes/no), DAS28-CRP (score), 25-OH-Vitamin D levels (no deficiency/subclinical/clinically relevant as defined by the university hospital laboratory), creatinine levels (mg/dl), seropositivity (yes/no; ‘yes’ includes the following groups: 1) positive for antibodies against cyclic citrullinated peptides [ACPA], 2) positive rheumatoid factor [RF] status, defined as IgA and/or IgM positivity; 3) double positive, defined as both positive ACPA and RF status), NSAID intake (yes/no), diabetes mellitus (type I, II, none), physical activity (1x/week, 2-3x/week, 4-6x/week, daily).

As effect modifiers, we assessed age (stratified arbitrarily into elderly [≥ 65 years] and non-elderly [< 65 years]) and sex (men, women).

### Statistical methods

All *p* values and confidence intervals were two-sided. We did not apply explicit adjustments for multiplicity, rather we kept the number of tests at a minimum (formal significance tests only for main comparisons). Descriptive statistics were used to summarize the collected relevant demographic variables and disease characteristics enabling an assessment of the balance across the two stratification variables (sex and age groups). Here, categorical data are described using numbers and percentages. Normally distributed continuous data are described using means and standard deviations, whereas continuous data that are skewed are described using medians and interquartile ranges.

The analyses were based on the “intention-to-monitor” population, i.e., based on all eligible individuals participating in the Rh-GIOP cohort fulfilling the eligibility criteria presented above. Missing data on outcomes and covariates were handled using multiple imputation (five imputations) under the assumption that data is “missing at random”. The primary analysis was rerun with as-observed data in a sensitivity analysis.

Separate multiple linear regression models were constructed, including as covariates the exposure and potential confounding variables mentioned above as well as an interaction between either GC and age, or GC and sex. Both crude (unadjusted) and adjusted estimates were reported. The primary analyses included minimum T-scores as outcome and current GC dose as exposure. Sensitivity analyses further investigated lumbar spine T-scores and total hip T-scores as outcomes, and cumulative GC dose and cumulative duration of GC as exposures, and respectively. A table listing all performed regression analyses was suggested during peer review and can be found in Additional file 2: Appendix B Table S7.

No formal sample size calculations were conducted. Rather, all enrolled patients whose data had been entered into the database at the time of database lock (March 16^th^, 2022) were analyzed.

R software (version 4.0.3) with packages *mice *[14] and *emmeans *[15] were used for the analyses.

## Results

### Patient characteristics

**Fig. 1:**
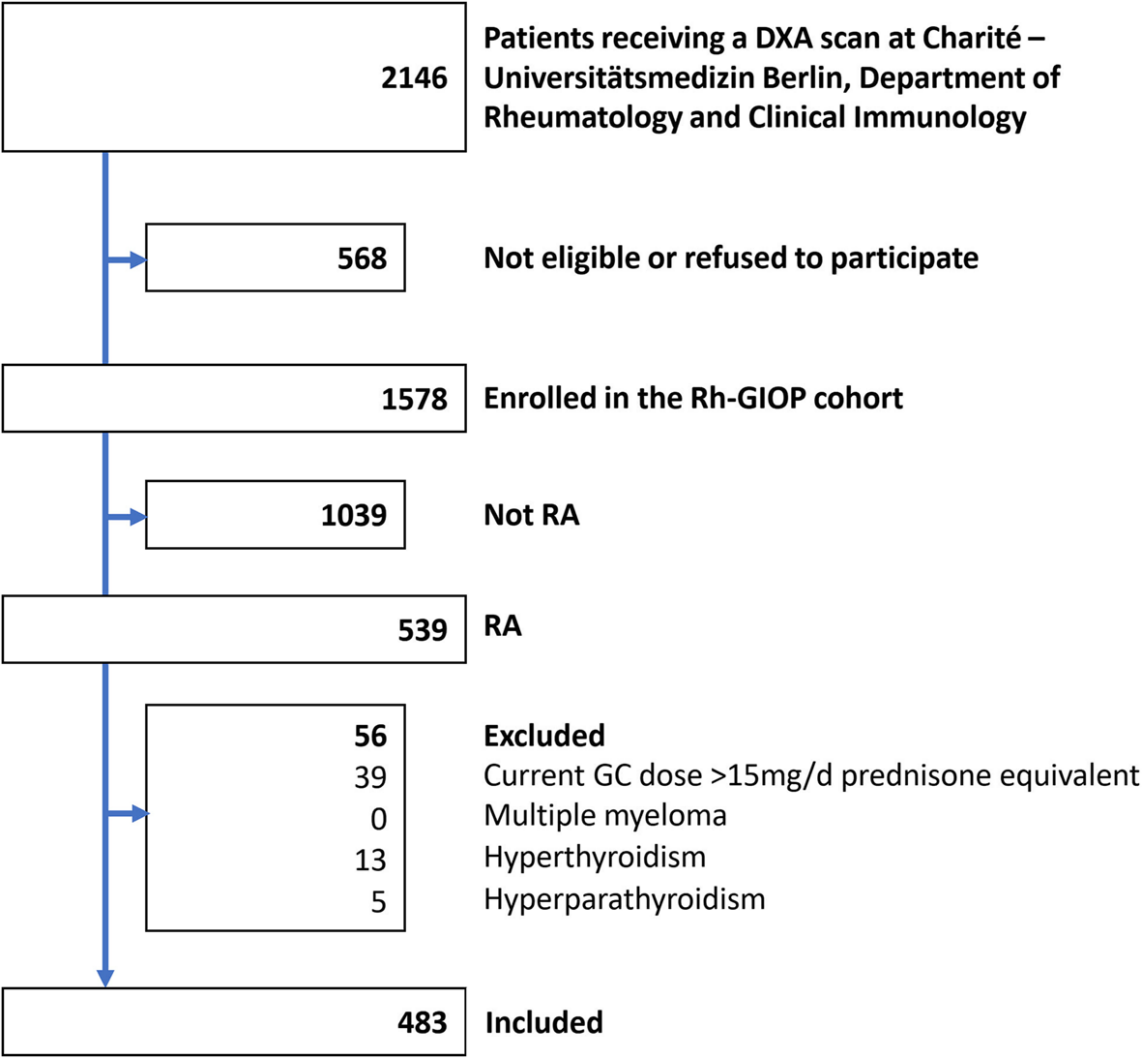
Study flow chart. DXA, dual x-ray absorptiometry; RA, rheumatoid arthritis; GC, glucocorticoid

Four hundred eighty-three patients with RA (mean age 64 ± 11.7 years, 80% female) enrolled in the Rh-GIOP cohort between September 2015 and March 2022 were analysed (Fig. 1). Most patients were affected by RA for more than ten years (median disease duration of 13.0 years). A median disease activity score-28 joints (DAS-28-CRP) of 2.6 (interquartile range 1.8–3.5) indicated low disease activity at the time of BMD measurement. Almost nine out of ten patients were taking a disease-modifying antirheumatic drug (DMARD) with conventional synthetic DMARDs being the most commonly used (65%), followed by biological DMARDs (38%). With regard to GCs, while 33% were not taking any, 32% were treated with a dose of 5 mg/d. Additional file 2: Appendix B Table S8, which was suggested during peer review, shows cumulative GC dose and duration of therapy of GC users and non-users: Current users had a higher cumulative dose and duration compared with non-users. About half of the patients had suffered any kind of (including both fragility and traumatic) non-vertebral fracture before enrollment, and almost 10% had experienced any kind of clinical vertebral fracture. 23% had OP by DXA (WHO definition of ≤ -2.5).

Disease activity measured by DAS-28 was similar across groups. Compared to women, men in this cohort had a higher concentration of C-reactive protein (4.6 vs. 2.1 mg/l). More than a third of women (35.8%), and less than a fifth of the men (18.9%), currently received no GC. Men also seemed slightly more likely to receive higher GC doses (> 7.5 mg/d) than women – 14.7% vs 10.1%, respectively. Regarding differences in RA treatment, one out of ten (10.1%) non-elderly patients received a targeted synthetic DMARD (tsDMARD), while the same was true for only 3.8% of the elderly population.

33.4% of women had a self-reported family history of OP, but only 15.9% of men. Vertebral fractures were about three times more frequent in the elderly population (14.5% vs 4.8%). Osteoporosis and anti-osteoporotic treatment were more frequent in the elderly group (28.9% vs 18.1% and 19.6% vs 6.9%, respectively). Further patient characteristics, stratified into men and women and elderly and non-elderly patients, can be found in Table 1. Overall, 5% of data points were missing, but only very little data was missing for the most relevant variables: age 0%, sex < 1%, current GC dose 0%, minimum T-score 1%.

**Table 1:** Patient characteristics

	**All (*n* = 483)**	**Men (*n* = 95)**	**Women (*n* = 388)**	**Elderly (*n* = 235)**	**Non-elderly (*n* = 248)**
**Age, years**	64.3 (11.7)	66.2 (11.2)	63.8 (11.7)	73.9 (6.0)	55.1 (7.7)
**Women, n (%)**	388 (80.3)	0 (0.0)	388 (100.0)	180 (76.6)	208 (83.9)
**DAS28-CRP, score, median (IQR)**	2.6 (1.8–3.5)	2.5 (1.8–3.3)	2.6 (1.7–3.5)	2.4 (1.7–3.2)	2.6 (1.9–3.8)
**Seropositive, n (%)**	328 (74.7)	59 (74.7)	269 (74.7)	150 (77.7)	178 (72.4)
**Disease duration, years, median (IQR)**	13.0 (9.0–21.0)	12.0 (8.0–19.0)	14.0 (9.0–22.0)	15.0 (9.0–24.0)	13.0 (8.0–19.0)
**Current GC dose, n (%)**^**a**^					
0 mg/d	157 (32.5)	18 (18.9)	139 (35.8)	81 (34.5)	76 (30.6)
> 0 to < 5 mg/d	97 (20.1)	22 (23.2)	75 (19.3)	51 (21.7)	46 (18.5)
5 mg/d	151 (31.3)	34 (35.8)	117 (30.2)	72 (30.6)	79 (31.9)
> 5 to ≤ 7.5 mg/d	25 (5.2)	7 (7.4)	18 (4.6)	9 (3.8)	16 (6.5)
> 7.5 mg/d	53 (11.0)	14 (14.7)	39 (10.1)	22 (9.4)	31 (12.5)
**Cumulative dose, g, median (IQR)**	9.5 (3.4–24.6)	10.4 (3.8–25.6)	9.4 (3.2–24.6)	9.2 (3.2–26.6)	9.7 (3.6–23.0)
**Cumulative duration of GC use, years, median (IQR)**	5.8 (2.0–13.0)	5.0 (2.5–12.1)	6.0 (2.0–13.0)	5.6 (2.0–14.0)	6.0 (2.0–11.2)
**DMARD use, n (%)**	421 (87.2)	82 (86.3)	339 (87.4)	207 (88.1)	214 (86.3)
csDMARDs	312 (64.6)	61 (64.2)	251 (64.7)	158 (67.2)	154 (62.1)
bDMARDs	183 (37.9)	34 (35.8)	149 (38.4)	81 (34.5)	102 (41.1)
tsDMARDs	34 (7.0)	6 (6.3)	28 (7.2)	9 (3.8)	25 (10.1)
**Alcohol consumption, n (%)**					
None	231 (48.5)	37 (38.9)	194 (50.9)	116 (50.7)	115 (46.6)
Irregular/infrequent	219 (46.0)	47 (49.5)	172 (45.1)	96 (41.9)	123 (49.8)
Occasional	23 (4.8)	9 (9.5)	14 (3.7)	16 (7.0)	7 (2.8)
Frequent	3 (0.6)	2 (2.1)	1 (0.3)	1 (0.4)	2 (0.8)
**Smoking, n (%)**					
Never	230 (48.0)	38 (40.0)	192 (50.0)	123 (53.0)	107 (43.3)
Former	165 (34.4)	36 (37.9)	129 (33.6)	86 (37.1)	79 (32.0)
Current	84 (17.5)	21 (22.1)	63 (16.4)	23 (9.9)	61 (24.7)
**Body Mass Index, kg/m**^**2**^	27.6 (5.4)	28.1 (3.5)	27.5 (5.8)	27.6 (5.2)	27.6 (5.7)
**C-reactive protein mg/l, median (IQR)**	2.3 (0.8–5.6)	4.6 (1.4–13.8)	2.1 (0.7–4.7)	2.6 (1.0–7.5)	1.7 (0.7–5.0)
**Vitamin D supplementation, n (%)**	403 (83.4)	80 (84.2)	323 (83.2)	200 (85.1)	203 (81.9)
**Calcium supplementation, n (%)**	33 (6.8)	9 (9.5)	24 (6.2)	14 (6.0)	19 (7.7)
**Serum 25-OH vitamin D3, n (%)**					
> 50 nmol/l	395 (87.4)	80 (87.9)	315 (87.3)	199 (90.5)	196 (84.5)
25–50 nmol/l	54 (11.9)	11 (12.1)	43 (11.9)	19 (8.6)	35 (15.1)
< 25 nmol/l	3 (0.7)	0 (0.0)	3 (0.8)	2 (0.9)	1 (0.4)
**Anti-osteoporotic therapy, n (%)**					
Bisphosphonates	63 (13.0)	11 (11.6)	52 (13.4)	46 (19.6)	17 (6.9)
Denosumab	17 (3.5)	1 (1.1)	16 (4.1)	14 (6.0)	3 (1.2)
Teriparatide	3 (0.6)	0 (0.0)	3 (0.8)	1 (0.4)	2 (0.8)
**Prior vertebral fracture, n (%)**^**b**^	46 (9.5)	11 (11.6)	35 (9.0)	34 (14.5)	12 (4.8)
**Prior non-vertebral fracture, n (%)**^**b**^	255 (52.8)	45 (47.4)	210 (54.1)	121 (51.5)	134 (54.0)
**Family history of osteoporosis, n (%)**	107 (30.1)	11 (15.9)	96 (33.4)	40 (24.5)	67 (34.7)
**Family history of osteoporotic fracture, n (%)**	46 (13.1)	3 (4.3)	43 (15.2)	20 (12.4)	26 (13.7)
**Minimum T-score**	-1.7 (1.1)	-1.6 (1.1)	-1.7 (1.1)	-1.9 (1.1)	-1.4 (1.1)
**Osteoporosis by DXA, n (%)**	113 (23.4)	21 (22.1)	92 (23.7)	68 (28.9)	45 (18.1)

*DAS28* disease activity score 28 joints, *GC* glucocorticoid, *DMARD* disease-modifying antirheumatic drugs, *DXA* dual x-ray absorptiometry

^a^Glucocorticoid doses were converted into prednisone equivalent doses

^b^Any fracture (including traumatic fractures). Numbers are n (%), mean (standard deviation) or median (interquartile range)

### Sex as a potential effect modifier

**Fig. 2:**
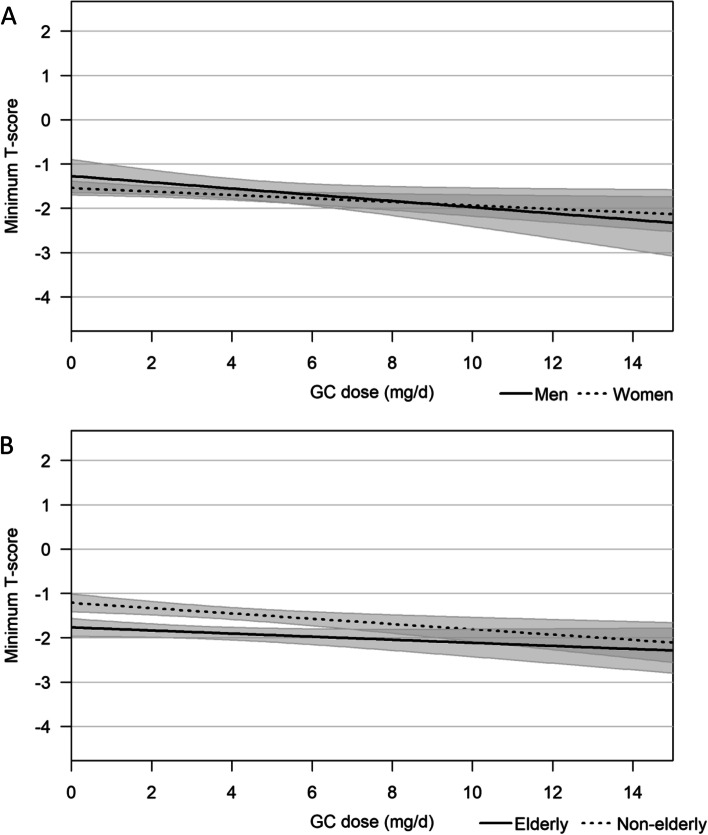
**A** and **B** Regression lines showing the association between current GC dose and minimum T-score for sex subgroups (**A**) and age subgroups (**B**). Shadows surrounding the regression lines are the respective 95% confidence intervals

Neither in crude nor in adjusted models did we find evidence of sex being an effect modifier regarding the effect of GCs on minimum T-scores (Fig. 2A and Table 2). The slope, i.e., the associated change in minimum T-score levels with 1 mg/d change in current GC dose, was -0.07 for men and -0.04 for women in both crude and adjusted models (*p* value for interaction = 0.437 and 0.410, respectively). In other words, the difference between men and women was not statistically significant. Using as-observed data instead of imputed data did not change the results of this analysis (Additional file 2: Appendix B Table S1). The results also remained consistent across all protocolized sensitivity analyses (Additional file 2: Appendix B Tables S2 and S3) that addressed cumulative GC dose and cumulative duration of GC use as secondary exposures of interest. Finally, the results were similar as well when looking at lumbar spine and total hip T-scores instead of minimum T-scores (Additional file 2: Appendix B Tables S4 and S5). Mean minimum T-scores across quartiles of cumulative GC dose stratified by age and sex were requested during peer review and are presented in the Additional file 2: Appendix B Table S6.

**Table 2:** Interaction between current GC dose and sex and current GC dose and age regarding minimum T-scores

**Overall**				
crude	–0.04 (0.02)			
adjusted	–0.04 (0.02)			
	**Men**	**Women**	**Difference**	***p* value**
crude	–0.07 (0.04)	–0.04 (0.02)	–0.03 (–0.11 to 0.04)	0.44
adjusted	–0.07 (0.03)	–0.04 (0.02)	–0.03 (–0.10 to 0.04)	0.41
	**Non–elderly**	**Elderly**		
crude	–0.06 (0.02)	–0.04 (0.02)	–0.02 (–0.08 to 0.03)	0.39
adjusted	–0.04 (0.02)	–0.03 (0.02)	–0.01 (–0.06 to 0.05)	0.77

Numbers are slope (standard error) except *p* value

### Age as a potential effect modifier

The second potential effect modifier evaluated was age. Again, in both crude and adjusted models, *p* values for interaction were not statistically significant (*p* value for interaction = 0.390 and 0.772), meaning that the effect of GCs on BMD was not statistically significantly different in elderly and non-elderly patients (Table 2). Once more, no evidence of interaction was found when choosing different GC-related exposures of interest (cumulative GC dose and cumulative duration of GC use; Additional file 2: Appendix B Tables S2 and S3) or different BMD outcomes (lumbar spine and total hip T-scores; (Additional file 2: Appendix B Tables S4 and S5). The results of our primary analysis did not change when using as observed data either (Additional file 2: Appendix B Table S1).

## Discussion

In this cross-sectional study of patients with RA, the association between GCs and BMD was not modified by age or sex. To our knowledge, this is the first study to investigate sex and age as potential effect modifiers regarding the association between GCs and bone health.

We live in an era of advancing personalization in medicine [16]. The ongoing discovery of biomarkers helps tailor the treatment of chronic diseases. In oncology, for example, the presence of the BCR-ABL mutation in chronic myeloid leukemia is an effect modifier regarding treatment with Imatinib – as a consequence, BCR-ABL-negative chronic myeloid leukemia needs to be treated with different drugs and yields other outcomes [17]. In the field of rheumatology, there are ongoing efforts to personalize treatment approaches as well. In rheumatoid arthritis, for example, treatment with rituximab or abatacept is more effective in ACPA positive patients [18]. However, effect modifiers need not always be the product of complex or costly diagnostics. Every single characteristic a patient possesses could theoretically be an effect modifier. Age and sex are two easily collected characteristics. If such characteristics are indeed found to impact treatment response or adverse event occurrence, practice change could follow quickly and without complicated implementation associated with elaborate tests.

GCs are still often used in RA. Especially in difficult-to-treat RA, GCs remain an important remedy in the rheumatologist’s armamentarium. However, especially with chronic use of higher dosages, they can cause a multitude of adverse events. OP was ranked one of the most feared GC-associated adverse events in a survey of both rheumatologists and patients with rheumatic diseases [19]. Furthermore, independently of their effects on BMD, GCs also seem to impact bone microarchitecture, which cannot be measured with 'regular’ bone densitometry. For example, patients taking GCs suffer from fractures occurring at higher BMD compared to control patients [20, 21]. However, in the study at hand, we did not plan to quantify the overall association between GCs and BMD or fractures as we have done so before [11].

Rheumatoid arthritis can occur at both young and old age (“late-onset RA”, also abbreviated “LORA”). These subsets of patients receive different treatments, differ in laboratory markers, and have differing disease outcomes [22–25]. OP is in general more common among elderly compared to younger patients, which is why we hypothesized that GCs might have different effects on BMD in these patient subsets. However, there was no significant interaction in any of our analyses. The results remained the same regardless of whether the main exposure of interest was current GC dose, cumulative GC dose, or cumulative duration of GC use. As described above, while comparing the elderly and non-elderly, the latter received tsDMARDs almost thrice as often. Concerning GC dosing, the non-elderly population received doses higher than 5 mg/d more often than the elderly, perhaps because of increased fear of adverse events in older individuals. Smoking was more common in the non-elderly population. Clinically relevant vitamin D deficiencies were overall rare among both groups, but subclinical deficiencies were observed twice as often in the non-elderly population.

Similar to the differences between young and old RA patients, women and men differ with regard to RA. The disease is more common in women with a ratio of about 3:1 [26], and some studies found that women are treated differently than men [26, 27]. In our study no differences were observed regarding treatment with DMARDs between both sexes, however, men were more likely to receive higher GC doses.

This study has some limitations. It is an observational study, of cross-sectional nature, conducted at a single center, and BMD is only a surrogate measure for OP and its consequence – fragility fractures. Fractures were not assessed in inferential statistical analyses because of low numbers. Interestingly, the overall prevalence of vertebral fractures was rather low compared to other studies in rheumatoid arthritis [28]. Furthermore, using 65 years as an age cut-off for dichotomization into elderly and non-elderly is arbitrary and other authors have recommended other cut-off values or refraining from such an arbitrary grouping in general [29, 30]. Another limitation is that higher-order interactions could not be assessed due to inadequate patient numbers for such analyses. Strengths include adjustment for a variety of confounders (including disease activity), measurement of BMD with the same equipment and operators across all participants, the use of several GC-related measures (current and cumulative dose and cumulative duration of GC use), and conduct according to a pre-specified statistical analysis plan.

## Conclusions

In summary, this study did not find age and sex to be effect modifiers with regard to the impact of GCs on BMD in patients with RA.

## Supplementary Information

**Additional file 1.**

**Additional file 2.**

## Data Availability

AP and FB are willing to examine all reasonable requests for data for a period of five years from publication.

## Abbreviations

RA: Rheumatoid arthritis
OP: Osteoporosis
BMD: Bone mineral density
GC: Glucocorticoid
