# Supplementary material for: Sex and age do not modify the association between glucocorticoids and bone mineral density in patients with rheumatoid arthritis: a cross-sectional study
Source: Arthritis Res Ther. 2023 Jun 7;25:98. doi: 10.1186/s13075-023-03083-x (PMC10246103; doi:10.1186/s13075-023-03083-x)
Supplement: Supplementary file 1 — Additional file 1. [file 13075_2023_3083_MOESM1_ESM.pdf]

May 6th, 2022

Interaction Between Glucocorticoids and Sex and Age in Bone Mineral Density Levels in Patients with Rheumatoid Arthritis: A Statistical Analysis Plan for a Cross-Sectional Study

Section 1: Administrative Information

Collaborators

Andriko Palmowski[1,2], Zhivana Boyadhzieva[1], Sabrina M Nielsen[2,3], Burkhard Muche[1], Sandra Hermann[1], Robin Christensen[2,3], Edgar Wiebe[1], Frank Buttgereit[1]

[1]Charité – Universitätsmedizin Berlin, Department of Rheumatology and Clinical Immunology

[2]Section for Biostatistics and Evidence-Based Research, The Parker Institute, Bispebjerg and Frederiksberg Hospital, Copenhagen, Denmark

[3]Research Unit of Rheumatology, Department of Clinical Research, University of Southern Denmark, Odense University Hospital, Odense, Denmark

Roles and responsibility

Andriko Palmowski: Junior lead author, concept & idea, acquisition of funding, data acquisition, first SAP draft, first manuscript draft

Zhivana Boyadzhieva: SAP draft, manuscript draft

Sabrina M Nielsen: Statistical analyst, performs all analyses, SAP draft, manuscript draft

Burkhard Muche: Expert advice, SAP draft, manuscript draft

Sandra Hermann: Expert advice, SAP draft, manuscript draft

Robin Christensen: Senior biostatistician, expert advice, SAP draft, manuscript draft

Edgar Wiebe: Cohort concept, data acquisition, data management, SAP draft, manuscript draft

Frank Buttgereit: Senior lead author, principal investigator, concept & idea, acquisition of funding, SAP draft, manuscript draft

Section 2: Introduction

Background

Rheumatoid arthritis (RA) is a chronic inflammatory autoimmune disease with potentially negative effects on the quality of life and life expectancy of affected patients. [1] Various disease-related factors are responsible for this, such as pain and reduced physical performance but also associated comorbidities such as osteoporosis (OP). [2]

Glucocorticoids (GCs) are widely used to treat RA and have potent anti-inflammatory effects. [3-8] According to current guidelines, GC therapy should primarily be used as bridging therapy. [9] This means that GCs should only be used for a short period of time to rapidly reduce disease activity at treatment initiation. Rapid tapering follows to avoid potential GC-associated side effects, including OP. However, some patients experience a flare-up of disease activity upon discontinuation of GCs. Others may achieve remission or low disease activity but remain chronically dependent on low-dose GCs. Although the role of low-dose GCs as long-term therapy for RA is considered controversial [10], real-life data shows that GCs are still often continued for years. [5, 11-13]

The benefits and risks of long-term therapy with GCs must be assessed accurately: It is known that GC-associated side effects increase in frequency and severity with higher dose and longer duration of therapy. One of the most worrying side effects of GC therapy is OP, as reported by both patients and rheumatologists. [14] RA regularly leads to a localized and generalized loss of bone substance and a reduction in bone quality; thus, OP is considered a complex comorbidity of the disease. [15] [16] The generally increased systemic inflammatory activity in RA and the upregulation of proinflammatory cytokines are associated with reduced bone formation and increased bone resorption. For example, Schett et al. found an increased risk of nontraumatic fractures at even minimal elevations in serum C-reactive protein. [17] GCs are also often cited a cause for the high prevalence of OP in RA patients. Namely, OP associated with GCs is thought to be the most common form of secondary OP. [18] GCs have a direct inhibitory effect on osteoblasts and additionally lead to osteoclast activation (at least initially), which increases bone loss. [18, 19] However, it should be taken into consideration that the anti-inflammatory effects of GCs can also have a bone-protective impact. [10] In addition, their analgesic and stiffness-reducing effects may lead to more exercise and thus have a positive impact on OP. [20]

Rationale for this study

To accurately weigh the risk-benefit ratio of GCs and specific anti-osteoporotic therapy in rheumatic diseases, many patient-specific factors need to be taken into account, such as nicotine abstinence and physical training (as protective factors) or inflammation and estrogen deficiency (as risk factors). [10] In the international widely used Fracture Risk Assessment Tool (FRAX) of the University of Sheffield, RA is listed as a risk factor, as is treatment with GCs. [21] However, a major weakness of this tool is that GC therapy is not differentiated – for example, multiple variables should be included such as current and cumulative dose – but GCs are only treated as a dichotomous variable ("yes/no"). There are some ideas on how to correct for GC dosages (also from the developers of FRAX),[22] but this was not directly imbedded in the FRAX online calculator.

The exact impact of GCs on the risk of OP in RA has not yet been adequately studied. GCs have long been viewed solely as risk factors. [23, 24] However, the evidence has mostly arisen from observational studies which are prone to "confounding by indication" [25]. Patients with higher disease activity are generally more likely to take higher doses of GCs. This makes it difficult to separate

the effects of high disease activity from the effects of GC therapy. While it is known that both factors influence BMD, the question of whether GCs in young and elderly patients, and in men and women, have a similar effect on osteoporosis, remains insufficiently answered. Performing an investigation to see if there are differences in those subpopulations could contribute to a more individualized approach in the therapy of RA in the future (“personalized medicine”).

Objectives

In this study, we aim to assess the impact of age (A) and sex (S) on the effects GC (G) exert on the dependent variable bone mineral density (BMD, *Y*) in patients with RA, i.e., we want to look for an interaction between age and GCs (A×G), and sex and GCs (S×G) while taking the main effects into consideration (A, S, and G, respectively). This will be done by analyzing cross-sectional baseline data from the Rh-GIOP cohort (https://clinicaltrials.gov/ct2/show/NCT02719314

Section 3: Study Methods

Trial design - Rh-GIOP Cohort

In 2015, the Rh-GIOP cohort study (Glucocorticoid-Induced Osteoporosis in Patients with chronic inflammatory Rheumatic Diseases or Psoriasis - Rh-GIOP) was initiated (registered at clinicaltrials.gov: NCT02719314; positive vote of the local ethics committee of Charité Universitaetsmedizin - Berlin: EA1/367/14]). RA patients at Charité - Universitätsmedizin Berlin who have a history of GC use and who have an indication for osteoporosis diagnostics (according to the German umbrella osteology association [DVO] guidelines) have been included in the study and were systematically investigated. We also included patients in control groups, e.g. patients without GCs and/or without an inflammatory rheumatic disease, and patients with psoriasis (without arthritis). In this study, we include a subpopulation of the Rh-GIOP cohort (see section 5: Trial population).

Various relevant variables are collected at each visit (**Box 1**). Thus, to our knowledge, Rh-GIOP is the largest prospective cohort study of osteoporosis in patients with rheumatic diseases worldwide in which such a large number of parameters relevant to bone health are specifically recorded over such a long time (and data collection is still ongoing). In a first large cross-sectional study of our extensive database, low GC doses of < 5mg/d prednisolone equivalent appear to be "relatively safe" for bone health (manuscript currently in peer-review).

Box 1. Parameters collected at each visit.

Demographics and general information	Age, sex, BMI, smoking status, type and frequency of sports activities, sunlight exposure, calcium intake, need for specific care, socioeconomic status
Description of GC therapy	Average daily dose, cumulative dose, duration of therapy
Description of underlying	Disease onset, current disease activity, disease-related comorbidities, patient-reported outcomes, earlier and current therapy with antirheumatic drugs
General bone-related parameters	Vitamin D and calcium supplements, treatment with anti-osteoporotic drugs, treatment with drugs with a known/possible effect on bone (i.e., proton pump inhibitors)
Clinical bone-related parameters	Osteoporosis/fractures in familial history, chair-raising test, frailty assessment, back pain, earlier vertebral and non-vertebral fractures during inadequate trauma, date of fracture, treatment of fractures, fracture sequelae, weight loss, height loss, previous falls, risk assessment of falls, menarche/menopause/pregnancy/lactation/early use of hormonal contraceptives
Technical bone-related parameters	routine laboratory tests (calcium, phosphate, vitamin D (1,25- and 25), iPTH, alkaline phosphatase, cross-links, etc.), BMD and T-score measured by DXA, TBS

BMI, Body-Mass-Index; BMD, Bone mineral density; GC, Glucocorticoid; iPTH, intact parathyroid hormone; DXA, Dual Energy X-ray Absorptiometry; TBS, Trabecular Bone Score

Sample size

For the present cross-sectional study, we will include all patients with a diagnosis of RA which are included until the database lock (performed on March 16th, 2022).

Section 4: Statistical principles

Confidence intervals and *P* values

All *P* values and confidence intervals will be two sided. We will not apply explicit adjustments for multiplicity, rather we will keep the number of tests at an absolute minimum (formal significance tests only for comparisons indicated in Table 2). Descriptive statistics will be used to summarize the collected relevant demographic variables and disease characteristics enabling an assessment of the balance across the two stratification variables (Sex and Age group). These data will be presented in the primary manuscript as outlined in **Table 1.** Categorical data will be described using numbers and percentages. Normally distributed continuous data will be described using means and SDs, whereas continuous data that are skewed will be described using medians and interquartile ranges. Statistical inference (i.e., from statistical tests) will only be reported for the analyses described in **Table 2**.

Analysis populations

The primary analyses will be based on the Intention to Monitor (ITM) population, i.e., based on the Full Analysis Set. Full Analysis Set will be defined as the eligible individuals agreeing to participate in

the Rh-GIOP study – patients with RA. Accordingly, participants agreeing to participate analyzed as part of the ITM population, irrespective of data availability or later withdrawal etc.

Section 5: Trial population

A flow diagram will be used to visualize and transparently report the progress through the phases of enrolment, data availability and subsequent data analysis; see **Figure 1**.

Screening data

In the Rh-GIOP cohort, we include all patients (provided they meet the eligibility criteria written down below) who have a physician referral for a bone density measurement. They are included consecutively if they give informed consent.

Eligibility

Eligible patients will have a physician diagnosis of RA. They need to be currently taking GCs or have a history of GC use. Patients need to have an indication for osteoporosis diagnostics (according to the German umbrella osteology association [DVO]). Breastfeeding and lactating women are excluded, as are patients unable to provide informed consent for any reason. The primary analyses will be based on all patients with an RA diagnosis. We will exclude patients with a potential other cause for a secondary OP, namely patients with a history of multiple myeloma, hyperthyroidism (including grave's disease), and hyperparathyroidism. Also, we will exclude patients with high GC doses that are not usually used for longer periods of time (i.e., >15mg/d prednisone equivalent).

Recruitment

Recruitment was taking place monocentric in the Department of Rheumatology and Clinical Immunology, Charité – Universitätsmedizin Berlin, a tertiary care university hospital. We include both in- and outpatients.

Baseline patient characteristics

We will report baseline patient characteristics in a Table 1, for the whole group of RA patients, and then stratified by sex (male vs. female) and age (elderly (≥65 years) vs. non-elderly (<65 years)).

Section 6: Analysis

Outcome definitions

The main outcome measure is the minimum T-score observed (selected from lumbar spine, total femur, or femoral neck, whichever is lowest). There will be one primary analysis investigating the interaction between current GC dose and sex, and between current GC dose and age.

Analysis methods

Separate multiple linear regression, with adjustment for the following known and suspected confounders (**Box 2**), as well as an interaction between either GC and age, or GC and sex. We will assess collinearity of the models by using collinearity diagnostics.

Box 1. Adjustment specifications for linear regression models.

**Main independent variable**
Exposure: GC[1]
**Potential confounders included for adjustment**
Age, years
Sex (men, women)
Smoking status (current, former, no smoking)
Body mass index, kg/m^2
Family history of osteoporotic fractures (yes/no)
Alcohol consumption (none, irregular/infrequent, occasional, frequent)
Health Assessment Questionnaire (HAQ)
Proton pump inhibitor use (yes/no)
Disease duration, years
Bisphosphonate use (yes/no)
Denosumab use (yes/no)
Teriparatide use (yes/no)
DAS28-CRP, score
25-OH-Vitamin D deficiency (no deficiency/subclinical/clinically relevant)
Creatinine levels, mg/dl
Seropositivity (yes/no)
NSAID intake (yes/no)
Diabetes Mellitus Type (type I, II, none)
Physical activity, (1x/week, 2-3x/week, 4-6x/week, daily)
[1]GC: Depending on the analysis, either current or cumulative GC dose or cumulative duration of GC use

Missing data

Missing data is for various reasons difficult to avoid. We will apply the analysis framework suggested by White et al (2011) in which missing data related to the ITM approach depend on making plausible assumptions about the missingness of the data and including all participants in subsequent sensitivity analyses.[26] Missing data on the outcomes and covariates will be handled using multiple imputation (5 imputations), as well as the observed dataset. This approach should be valid assuming the data is Missing At Random (MAR).

De-confounding variables

Confounding variables are independent variables other than the exposure variable (GC) that are correlated to the outcome of the study. Unaccounted for confounding variables prevent us from measuring the true impact the GC exposure has on an outcome.

Thus, adjustment methods attempt to correct for the assignment mechanism (exposure to higher GC dose) by finding control units similar to treatment units. Prespecifying possible de-confounders, we will use the following pragmatic definition of what makes a confounding variable (*C*):

- The Covariate (*C*) is an ancestor (cause) of the outcome (*Y*)

- The Covariate (*C*) probably causes the exposure (*X*; GC group)
- The Covariate (*C*) is not a descendant (effect) of the exposure (*X*) or outcome (*Y*)

(De-) Confounding factors: The challenge with observational data is that treatments are not applied randomly, leading to selection bias and confounding variables. We will adjust for potential confounders listed in box 2 above.

Additional analyses

Sensitivity analyses:

Instead of independent variable current GC dose: cumulative GC dose / duration of GC therapy; then interaction term for sex*cumulative dose, age*cumulative dose, sex*duration of GC therapy, and age*duration of GC therapy, respectively.

Instead of dependent variable minimum T-Score: lumbar spine T-Score, and total hip T-score (of both right and left femur).

Furthermore, the primary analyses will also be conducted based on available data ('as observed').

Statistical software

R software or similar will be used for all analyses.

Section 7: Manuscript Outline

Figure 1: Flow diagram

Table 1: Patient Characteristics
(for all patients, then separately for women and men, and separately for elderly (≥65 years) vs. non-elderly (<65 years).

Figure 2: Scatterplot showing the association between current GC dose and minimum T-score for sex subgroups (panel a) and age subgroups (panel b), (y-axis: minimum T-score; x-axis: current GC dose; color coding of dots: sex or age subgroups; separate regression lines for sex or age subgroups with respective 95% confidence intervals)

Table 2: Association between current GC dose (linear) and minimum T-score for sex and age subgroups (Results of multiple regression with minimum T-score as the dependent variable, current GC dose and with and without above-mentioned confounders as independent variables [i.e., adjusted and crude, respectively], and an interaction between sex and current GC dose and age and current GC dose; multiple imputation will be applied)

Appendix Table S1: Association between current GC dose and minimum T-score for sex and age subgroups ('as observed')

Appendix Table S2: Association between cumulative GC dose and minimum T-score for sex and age subgroups

Appendix Table S3: Association between duration of GC use and minimum T-score for sex and age subgroups

Appendix Table S4: Association between current GC dose and lumbar spine T-score for sex and age subgroups

Appendix Table S5: Association between current GC dose and total hip (of both right and left femur) T-score for sex and age subgroups

Figure 1: Flow diagram

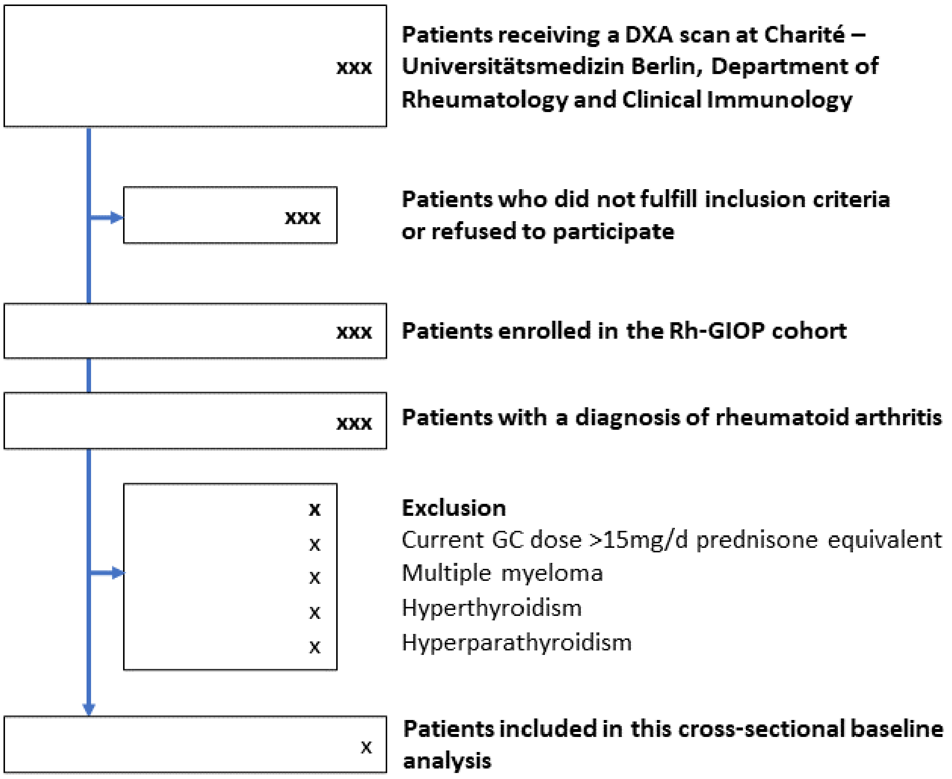

Table 1: Patient characteristics

		Sex		Age groups	
	**All (n=XXX)**	**Men (n=XXX)**	**Women (n=XXX)**	**Elderly (n=XXX)**	**Non-elderly (n=XXX)**
Age, years					
Women, n (%)					
DAS-28, score					
Disease duration, years					
Current GC dose mg/d					
0 mg/d, n (%)					
0.01 – <5 mg/d, n (%)					
5mg/d, n (%)					
5.01 – 7.5mg/d, n (%)					
> 7.5mg/d, n (%)					
Cumulative dose, g					
Cumulative duration of GC use, years					
DMARD use, n (%)					
csDMARDs, n (%)					
bDMARDs, n (%)					
tsDMARDs, n (%)					
Alcohol consumption					
None, n (%)					
Irregular/infrequent, n (%)					
Occasional, n (%)					
Frequent, n (%)					
Smoking					
Never, n (%)					
Former, n (%)					
Current, n (%)					
Body Mass Index, kg/m^2					
C-reactive protein mg/l					
Vitamin D supplementation, n (%)					
Calcium supplementation, n (%)					
25-OH vitamin D3 deficiency					
No deficiency (>50 nmol/l), n (%)					
Subclinical (25-50 nmol/l), n (%)					
Clinically relevant (<25 nmol/l), n (%)					
Anti-osteoporotic therapy					
Bisphosphonates, n (%)					
Denosumab, n (%)					
Teriparatide, n (%)					
Prior vertebral fracture, n (%)					
Prior non-vertebral fracture, n (%)					
Family history of osteoporosis, n (%)					
Family history of osteoporotic fracture, n (%)					
Min T-score					
Lumbar spine T-score					
Total hip T-score					
Osteoporosis by DXA, n (%)					
Seropositive, n (%)					

All values are mean (SD) unless otherwise stated.

Figure 2: Scatterplot showing the association between current GC dose and minimum T-score for sex subgroups (panel a, example below), age subgroups (panel b)

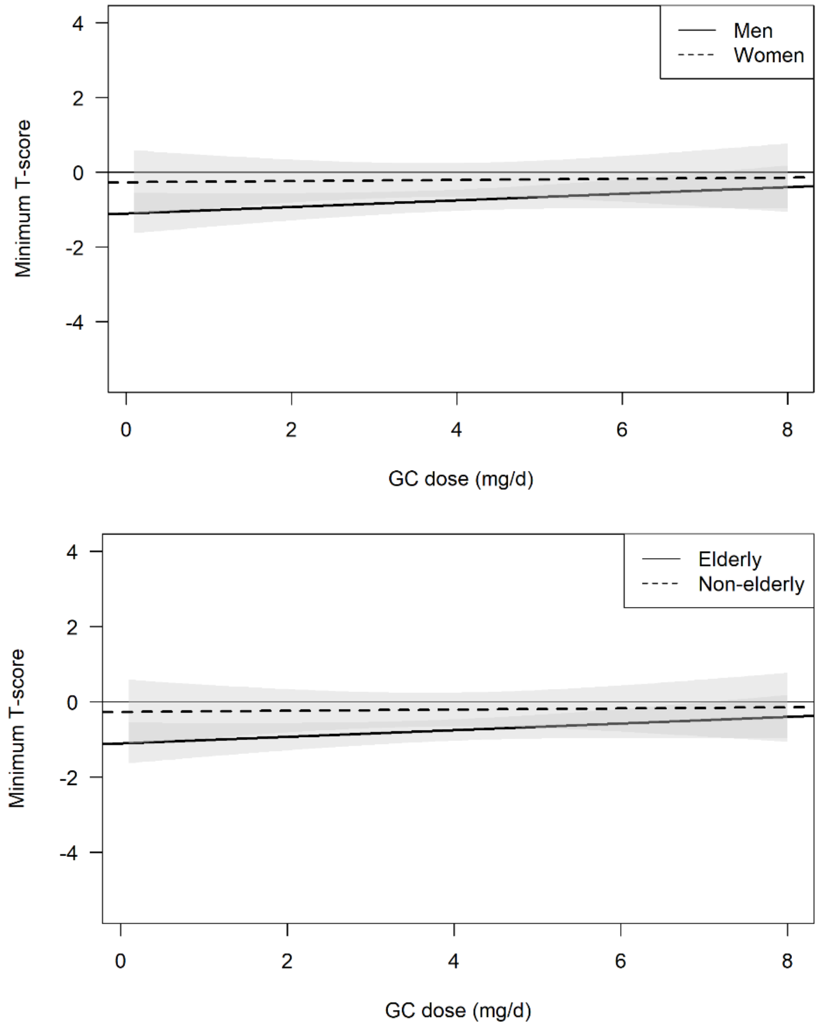

Table 2: Association between current GC dose and minimum T-score for sex and age subgroups

	Crude			Adjusted*		
	**β (SE)**	**Dif. (95%CI)**	**P value**	**β (SE)**	**Dif. (95%CI)**	**P value**
Overall	X (X)			X (X)		
Sex		X (X to X)	X		X (X to X)	X
Men	X (X)			X (X)		
Women	X (X)			X (X)		
Age groups		X (X to X)	X		X (X to X)	X
Elderly	X (X)			X (X)		
Non-elderly	X (X)			X (X)		

*adjusted for the covariates shown in *Box 2* above.

References

1. Matcham, F., et al., *The impact of rheumatoid arthritis on quality-of-life assessed using the SF-36: a systematic review and meta-analysis.* Semin Arthritis Rheum, 2014. **44**(2): p. 123-30.
2. Gullick, N.J. and D.L. Scott, *Co-morbidities in established rheumatoid arthritis.* Best Pract Res Clin Rheumatol, 2011. **25**(4): p. 469-83.
3. Cutolo, M., S. Paolino, and E. Gotelli, *Glucocorticoids in rheumatoid arthritis still on first line: the reasons.* Expert Rev Clin Immunol, 2021. **17**(5): p. 417-420.
4. Hua, C., F. Buttgereit, and B. Combe, *Glucocorticoids in rheumatoid arthritis: current status and future studies.* RMD Open, 2020. **6**(1).
5. Hanly, J.G. and L. Lethbridge, *Use of Disease-modifying Antirheumatic Drugs, Biologics, and Corticosteroids in Older Patients With Rheumatoid Arthritis Over 20 Years.* J Rheumatol, 2021. **48**(7): p. 977-984.
6. Hua, C., F. Buttgereit, and B. Combe, *Glucocorticoids in rheumatoid arthritis: current status and future studies.* RMD Open, 2020. **6**(1): p. e000536.
7. Spies, C.M., et al., *Pharmacology of glucocorticoids in rheumatoid arthritis.* Curr Opin Pharmacol, 2010. **10**(3): p. 302-7.
8. Buttgereit, F., *Views on glucocorticoid therapy in rheumatology: the age of convergence.* Nature Reviews Rheumatology, 2020. **16**(4): p. 239-246.
9. Smolen, J.S., et al., *EULAR recommendations for the management of rheumatoid arthritis with synthetic and biological disease-modifying antirheumatic drugs: 2019 update.* Ann Rheum Dis, 2020. **79**(6): p. 685-699.
10. Buttgereit, F., *Views on glucocorticoid therapy in rheumatology: the age of convergence.* Nat Rev Rheumatol, 2020. **16**(4): p. 239-246.
11. Boers, M. and T. Pincus, *Long-term Glucocorticoid Use in Rheumatoid Arthritis.* J Rheumatol, 2021. **48**(8): p. 1342.
12. Roubille, C., et al., *Seven-year tolerability profile of glucocorticoids use in early rheumatoid arthritis: data from the ESPOIR cohort.* Ann Rheum Dis, 2017. **76**(11): p. 1797-1802.
13. Albrecht, K., et al., *[Clinical remission in rheumatoid arthritis. Data from the early arthritis cohort study CAPEA].* Z Rheumatol, 2016. **75**(1): p. 90-6.
14. van der Goes, M.C., et al., *Patient and rheumatologist perspectives on glucocorticoids: an exercise to improve the implementation of the European League Against Rheumatism (EULAR) recommendations on the management of systemic glucocorticoid therapy in rheumatic diseases.* Ann Rheum Dis, 2010. **69**(6): p. 1015-21.
15. Raterman, H.G., I.E. Bultink, and W.F. Lems, *Osteoporosis in patients with rheumatoid arthritis: an update in epidemiology, pathogenesis, and fracture prevention.* Expert Opin Pharmacother, 2020. **21**(14): p. 1725-1737.

16. Llorente, I., et al., *Osteoporosis in Rheumatoid Arthritis: Dangerous Liaisons.* Front Med (Lausanne), 2020. **7**: p. 601618.
17. Schett, G., et al., *High-sensitivity C-reactive protein and risk of nontraumatic fractures in the Bruneck study.* Arch Intern Med, 2006. **166**(22): p. 2495-501.
18. Canalis, E., et al., *Glucocorticoid-induced osteoporosis: pathophysiology and therapy.* Osteoporos Int, 2007. **18**(10): p. 1319-28.
19. Buckley, L. and M.B. Humphrey, *Glucocorticoid-Induced Osteoporosis.* N Engl J Med, 2018. **379**(26): p. 2547-2556.
20. Wysham, K.D., J.F. Baker, and D.M. Shoback, *Osteoporosis and fractures in rheumatoid arthritis.* Curr Opin Rheumatol, 2021. **33**(3): p. 270-276.
21. Centre for Metabolic Bone Diseases, U.o.S., UK. *Fracture Risk Assessment Tool (FRAX).* 25.02.2022]; Available from: https://www.sheffield.ac.uk/FRAX/tool.aspx?country=9.
22. Kanis, J.A., et al., *Guidance for the adjustment of FRAX according to the dose of glucocorticoids.* Osteoporos Int, 2011. **22**(3): p. 809-16.
23. Van Staa, T.P., et al., *Use of oral corticosteroids and risk of fractures.* J Bone Miner Res, 2000. **15**(6): p. 993-1000.
24. van Staa, T.P., H.G. Leufkens, and C. Cooper, *The epidemiology of corticosteroid-induced osteoporosis: a meta-analysis.* Osteoporos Int, 2002. **13**(10): p. 777-87.
25. Boers, M., *Observational studies on glucocorticoids are harmful!* Lupus Sci Med, 2017. **4**(1): p. e000219.
26. White, I.R., et al., *Strategy for intention to treat analysis in randomised trials with missing outcome data.* Bmj, 2011. **342**: p. d40.
