# Supplementary material for: Sex and age do not modify the association between glucocorticoids and bone mineral density in patients with rheumatoid arthritis: a cross-sectional study
Source: Arthritis Res Ther. 2023 Jun 7;25:98. doi: 10.1186/s13075-023-03083-x (PMC10246103; doi:10.1186/s13075-023-03083-x)
Supplement: Supplementary file 2 — Additional file 2. [file 13075_2023_3083_MOESM2_ESM.pdf]

Appendix B: Tables

Sex and Age Do Not Modify the Association Between Glucocorticoids and Bone Mineral Density in Patients with Rheumatoid Arthritis: A Cross-Sectional Study

Andriko Palmowski[1,2], Zhivana Boyadzhieva[1], Sabrina M Nielsen[2,3], Burkhard Muche[1], Sandra Hermann[1], Maarten Boers[4], Henning Bliddal[2], Robin Christensen[2,3], Edgar Wiebe[1], Frank Buttgereit[1]

[1]Charité – Universitätsmedizin Berlin, Department of Rheumatology and Clinical Immunology

[2]Section for Biostatistics and Evidence-Based Research, The Parker Institute, Bispebjerg and Frederiksberg Hospital, Copenhagen, Denmark

[3]Research Unit of Rheumatology, Department of Clinical Research, University of Southern Denmark, Odense University Hospital, Odense, Denmark

[4] Department of Epidemiology & Data Science, Amsterdam University Medical Centers, Vrije Universiteit, Amsterdam, Netherlands

Corresponding Author

Dr. Andriko Palmowski, andriko.palmowski@charite.de

Table S1. Interaction between current GC dose and sex and current GC dose and age regarding minimum T-scores with as observed data.

		Crude			Adjusted	
	beta (SE)	Dif (95%CI)	P value	beta (SE)	Dif (95%CI)	P value
**Overall**	-0.045 (0.015)			-0.004 (0.040)		
**Sex**		-0.029 (-0.105 to 0.047)	0.453		-0.170 (-0.413 to 0.073)	0.167
**Men**	-0.070 (0.035)			-0.158 (0.118)		
**Women**	-0.041 (0.017)			0.012 (0.042)		
**Age**		-0.025 (-0.083 to 0.032)	0.386		0.011 (-0.165 to 0.188)	0.899
**Non-elderly**	-0.061 (0.020)			0.020 (0.048)		
**Elderly**	-0.036 (0.022)			0.009 (0.074)		

Table S2. Interaction between cumulative GC dose and sex and cumulative GC dose and age regarding minimum T-scores.

	Crude			Adjusted		
	**beta (SE)**	**Dif (95%CI)**	**P value**	**beta (SE)**	**Dif (95%CI)**	**P value**
**Overall**	-0.005 (0.002)			-0.004 (0.003)		
**Sex**		-0.005 (-0.017 to 0.007)	0.428		-0.006 (-0.017 to 0.006)	0.283
**Men**	-0.009 (0.006)			-0.009 (0.006)		
**Women**	-0.004 (0.002)			-0.003 (0.003)		
**Age**		-0.005 (-0.013 to 0.003)	0.267		-0.004 (-0.013 to 0.004)	0.358
**Non-elderly**	-0.007 (0.003)			-0.006 (0.003)		
**Elderly**	-0.002 (0.003)			-0.002 (0.003)		

Table S3. Interaction between cumulative duration of GC use and sex and cumulative duration GC use and age regarding minimum T-scores.

	Crude			Adjusted		
	**beta (SE)**	**Dif (95%CI)**	**P value**	**beta (SE)**	**Dif (95%CI)**	**P value**
**Overall**	-0.011 (0.005)			-0.010 (0.008)		
**Sex**		-0.012 (-0.039 to 0.016)	0.389		-0.013 (-0.037 to 0.012)	0.330
**Men**	-0.021 (0.013)			-0.020 (0.014)		
**Women**	-0.009 (0.006)			-0.008 (0.009)		
**Age**		-0.010 (-0.031 to 0.011)	0.356		-0.018 (-0.039 to 0.004)	0.066
**Non-elderly**	-0.016 (0.008)			-0.023 (0.010)		
**Elderly**	-0.006 (0.007)			-0.005 (0.010)		

Table S4. Interaction between current GC dose and sex and current GC dose and age regarding lumbar spine T-scores.

	Crude			Adjusted		
	**beta (SE)**	**Dif (95%CI)**	**P value**	**beta (SE)**	**Dif (95%CI)**	**P value**
**Overall**	-0.013 (0.019)			-0.012 (0.020)		
**Sex**		-0.018 (-0.121 to 0.084)	0.631		-0.019 (-0.122 to 0.085)	0.631
**Men**	-0.033 (0.047)			-0.027 (0.048)		
**Women**	-0.015 (0.022)			-0.009 (0.022)		
**Age**		-0.047 (-0.124 to 0.031)	0.198		-0.022 (-0.098 to 0.054)	0.475
**Non-elderly**	-0.036 (0.026)			-0.012 (0.027)		
**Elderly**	0.011 (0.029)			0.010 (0.029)		

Table S5. Interaction between current GC dose and sex and current GC dose and age regarding total hip T-scores.

	Crude			Adjusted		
	**beta (SE)**	**Dif (95%CI)**	**P value**	**beta (SE)**	**Dif (95%CI)**	**P value**
**Overall**	-0.046 (0.015)			-0.049 (0.016)		
**Sex**		-0.022 (-0.101 to 0.056)	0.527		-0.015 (-0.083 to 0.054)	0.695
**Men**	-0.066 (0.035)			-0.061 (0.032)		
**Women**	-0.044 (0.018)			-0.046 (0.018)		
**Age**		-0.016 (-0.074 to 0.042)	0.596		0.008 (-0.044 to 0.059)	0.795
**Non-elderly**	-0.058 (0.020)			-0.035 (0.020)		
**Elderly**	-0.042 (0.022)			-0.043 (0.022)		

Table S6. Mean minimum T-scores across quartiles of cumulative GC dose stratified by age and sex.

	≥65 years	<65 years	male	female
**1st quartile of cumulative GC dose**	-1.88 (1.06)	-1.23 (1.22)	-1.60 (1.09)	-1.55 (1.21)
**2nd quartile of cumulative GC dose**	-1.71 (1.24)	-1.37 (1.22)	-1.52 (1.23)	-1.53 (1.25)
**3rd quartile of cumulative GC dose**	-1.96 (0.89)	-1.56 (0.94)	-1.49 (0.89)	-1.80 (0.95)
**4th quartile of cumulative GC dose**	-2.00 (1.05)	-1.64 (1.01)	-1.85 (1.05)	-1.82 (1.05)

Table S7. Regression equations of all analyses (incl. sensitivity).

Data Type		Regression Equation	Table with Results
Imputed data	1)	Min T-score ~ Current GC dose*sex	Table 2
	2)	Min T-score ~ Current GC dose*age	Table 2
	3)	Min T-score ~ Current GC dose*sex + confounders	Table 2
	4)	Min T-score ~ Current GC dose*age + confounders	Table 2
	5)	Lumbar spine T-score ~ Current GC dose*sex	Table S4
	6)	Lumbar spine T-score ~ Current GC dose*age	Table S4
	7)	Lumbar spine T-score ~ Current GC dose*sex + confounders	Table S4
	8)	Lumbar spine T-score ~ Current GC dose*age + confounders	Table S4
	9)	Total hip T-score ~ Current GC dose*sex	Table S5
	10)	Total hip T-score ~ Current GC dose*age	Table S5
	11)	Total hip T-score ~ Current GC dose*sex + confounders	Table S5
	12)	Total hip T-score ~ Current GC dose*age + confounders	Table S5
	13)	Min T-score ~ Cumulative GC dose*sex	Table S2
	14)	Min T-score ~ Cumulative GC dose*age	Table S2
	15)	Min T-score ~ Cumulative GC dose*sex + confounders	Table S2
	16)	Min T-score ~ Cumulative GC dose*age + confounders	Table S2
	17)	Min T-score ~ Cumulative GC use duration*sex	Table S3
	18)	Min T-score ~ Cumulative GC use duration*age	Table S3
	19)	Min T-score ~ Cumulative GC use duration*sex + confounders	Table S3
	20)	Min T-score ~ Cumulative GC use duration*age + confounders	Table S3
As-observed data	21)	Min T-score ~ Current GC dose*sex	Table S1
	22)	Min T-score ~ Current GC dose*age	Table S1
	23)	Min T-score ~ Current GC dose*sex + confounders	Table S1
	24)	Min T-score ~ Current GC dose*age + confounders	Table S1

Confounders are listed in the main manuscript.

Table S8. Cumulative GC dose and duration of therapy of GC users and non-users.

	Currently on GCs	Currently not on GCs
**Cumulative GC dose, g prednisone equivalent**	12.6 (4.8-28.2)	4.6 (1.8-15.2)
**Cumulative duration of GC therapy, years**	7.3 (3.0-14.4)	2.6 (1.0- 8.2)

Numbers are median (interquartile range).
